# The lncRNA *hsrω* regulates arginine dimethylation of human FUS to cause its proteasomal degradation in *Drosophila*

**DOI:** 10.1242/jcs.236836

**Published:** 2019-10-15

**Authors:** Luca Lo Piccolo, Hideki Mochizuki, Yoshitaka Nagai

**Affiliations:** 1 Department of Neurotherapeutics, Osaka University Graduate School of Medicine, 2-2 Yamadaoka, Suita, Osaka 565-0871, Japan; 2 Department of Neurology, Osaka University Graduate School of Medicine, 2-2 Yamadaoka, Suita, Osaka 565-0871, Japan

**Keywords:** lncRNAs, Arginine dimethylation, Post-translation modification, RBP homeostasis, FUS toxicity, FUS cellular localisation, Neurodegeneration, *Drosophila*

## Abstract

Long non-coding RNAs (lncRNAs) have structural and regulatory effects on RNA-binding proteins (RBPs). However, the mechanisms by which lncRNAs regulate the neurodegenerative-causative RBP like FUS protein remain poorly understood. Here, we show that knockdown of the *Drosophila* lncRNA *hsrω* causes a shift in the methylation status of human FUS from mono- (MMA) to di-methylated (DMA) arginine via upregulation of the arginine methyltransferase 5 (PRMT5, known as ART5 in flies). We found this novel regulatory role to be critical for FUS toxicity since the PRMT5-dependent dimethylation of FUS is required for its proteasomal degradation and causes a reduction of high levels of FUS. Moreover, we show that an increase of FUS causes a decline of both *PRMT1* (known as ART1 in flies) and *PRMT5* transcripts, leading to an accumulation of neurotoxic MMA-FUS. Therefore, overexpression of either PRMT1 or PRMT5 is able to rescue the FUS toxicity. These results highlight a novel role of lncRNAs in post-translation modification (PTM) of FUS and suggest a causal relationship between lncRNAs and dysfunctional PRMTs in the pathogenesis of FUSopathies.

## Abstract (precis)

**Summary:** The lncRNA *hsrω* regulates the arginine methyltransferases type I and II to modify the human FUS RNA-binding protein, recombinantly expressed in flies, in a fashion that controls both its cellular localization and homeostasis.

## INTRODUCTION

Long non-coding RNAs (lncRNAs) are transcripts longer than 200 nucleotides found throughout the cell that lack protein-coding function (Lo Piccolo, 2018; Sun et al., 2018). It has been revealed nuclear-located lncRNAs form several complexes with structural and regulatory roles that allow gene organisation and control transcription (i.e. chromosome scaffolding, chromatin remodeling, alternative splicing, epigenetic control of transcription etc.) (Chujo and Hirose, 2017; Chujo et al., 2016; Nakagawa et al., 2018). Overall, the most common mechanism of action for lncRNAs is likely by interacting with and regulating the activity of RNA-binding proteins (RBPs) (Cui et al., 2018; Lan et al., 2018; Lee et al., 2016; Tripathi et al., 2010). Furthermore, lncRNA-interacting RBPs play fundamental cellular roles and their dysfunction leads to severe impairment of RNA processing in diverse pathological conditions.

Some nuclear lncRNAs show the ability to bind RNA-binding proteins (RBPs) containing a prion-like domain (PLD) and organize them into specific membrane-lacking organelles, such as the nuclear bodies (NBs) (Chujo et al., 2016). Complex eukaryotic cells carry several NBs with distinct functions (Platani and Lamond, 2004), such as paraspeckles, which control gene expression (Prasanth et al., 2005), and nuclear stress bodies, which are involved in reprogramming of gene expression upon stress (Jolly et al., 2004).

FUS is an RBP belonging to the FUS/TLS, EWS and TAF15 (FET) protein family, which functions by binding a large array of nucleic acids, including lncRNAs, and is involved in many aspects of RNA processing and DNA repair (Schwartz et al., 2015). Abnormal cytoplasmic localization or excessive levels of FUS are associated with a spectrum of neurodegenerative diseases, including amyotrophic lateral sclerosis (ALS) and frontotemporal lobar degeneration (FTLD) (Dini Modigliani et al., 2014; Doi et al., 2010; Naumann et al., 2018; Neumann et al., 2009; Sabatelli et al., 2013; Woulfe et al., 2010). Interestingly, some studies have shown that diverse nuclear lncRNAs are mis-expressed in FUS-ALS/FTLD suggesting that they have a close link with the occurrence of these pathologies (An et al., 2019; Gagliardi et al., 2018; Lourenco et al., 2015). However, whether and how the lncRNAs contribute to such disease still needs further elucidation.

Despite the recent progress, the functions of the large majority of lncRNAs and how they modulate the activity of RBPs still remain unknown (Kopp and Mendell, 2018). Since the lncRNAs are generally expressed at low levels and are poorly conserved, investigating the molecular mechanisms by which they act is tremendously challenging. Besides a poor nucleotide conservation among different species, some lncRNAs do in fact seem to retain similar functions. For instance, the human architectural lncRNA *SatIIIB*, which organizes the nuclear stress bodies, has been proposed to be the functional homolog of *Drosophila* nuclear lncRNA *hsrω* (Jolly and Lakhotia, 2006).

The lncRNA *hsrω* is known to interact with and organize several RBPs like the *Drosophila* FUS (Cabeza), TDP-43 (TBPH), hnRNPAB (Squid) and hnRNPA2B1 (Hrb87F) to form the ω-speckles, a specialized nuclear compartment that is functionally important; flies that are null for both copies of *hsrω* exhibit severe dysfunctions in RNA processing and chromatin structure, which causes lethality (Jolly and Lakhotia, 2006; Lakhotia and Sharma, 1996; Lo Piccolo et al., 2017a, 2018; Ray et al., 2019; Ray and Lakhotia, 1998). Together, with such a critical structural role, *hsrω* also regulates the activity of a large variety of proteins, including the histone acetyltransferase CBP, the chromatin remodeler ISWI and heterochromatin protein 1 (HP1) (Lakhotia et al., 2012; Mallik and Lakhotia, 2010; Onorati et al., 2011).

Given the many functions of lncRNAs and how they modulate the neurodegenerative-causative RBPs are poorly characterized, we generated new *in vivo* models to investigate the mechanism by which the nuclear *hsrω* functionally interacts with human FUS (herein recombinantly expressed human FUS is denoted as FUS) in *Drosophila*.

Interestingly, we found that the lncRNA *hsrω* modulates the regulation of arginine methylation of FUS. Mainly, we herein show that knocking down the nuclear *hsrω* transcript causes an upregulation of the arginine methyltransferase type II DART5 (the *Drosophila* homolog of PRMT5, known as ART5 in flies and denoted DART5 herein), which in turn modifies human FUS in a fashion that promotes its proteasomal degradation and eventually leads to a strong decline of the abundance of FUS and its associated toxicity. These results reveal a novel regulatory role of *hsrω* wherein it can control the post-translational modification of FUS and provide insight into how a nuclear lncRNA modulates the activity of an ALS/FTLD-causative RBP.

## RESULTS

### The lncRNA *hsrω* is a potent modifier of FUS

**Fig. 1.**
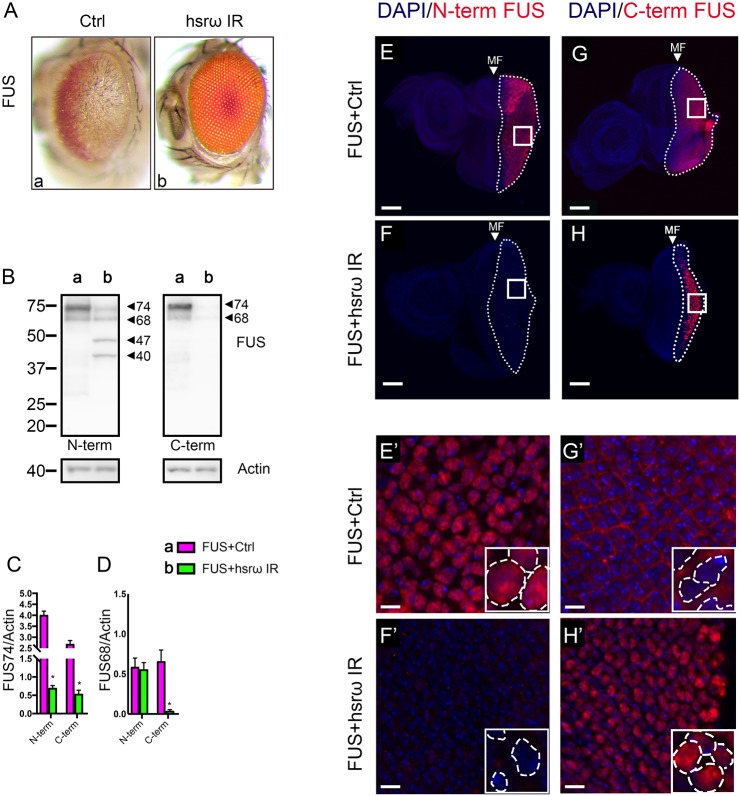
**Depletion of *hsrω* shows diverse effects on FUS.** (A) Light microscopy images of external eye surface of flies of genotype (a) *GMR-GAL4/+;* UAS*-FUS/+;* UAS*-GFP IR/+* (FUS+Ctrl) and (b) *GMR-GAL4/+;* UAS*-FUS/+;*UAS*-hsrω IR/+* (FUS+hsrω IR) raised at 28°C. (B–D) Total protein was extracted from adult heads and FUS expression was assayed by western blot analysis with anti-N-terminus (left panel) and anti-C-terminus (right) FUS IgG antibodies, respectively. Actin was loaded as internal control to quantify the relative abundance of the major FUS bands (74 kDa, FUS74; 68 kDa, FUS68). Two extra bands were detected by the anti-N-terminus FUS IgG antibody (47 kDa, FUS47; 40 kDa, FUS40, respectively). Statistical analysis was performed on three independent western blot experiments. *P<0.05. (E–H′) FUS immunohistochemistry was conducted using dissected brains of larvae at third stage of development (L3) raised at 28°C. The dotted lines mark the posterior region of the eye disc, which accordingly is the area of UAS expression driven by GMR. The small white squares (E–H) define the magnified areas shown in E′–H′. Insets in E′–H′ represent areas selected for higher magnification. Dotted in insets lines mark the area of nuclei. DAPI staining of DNA is shown in blue. FUS staining is shown in red. Both anti-N-terminus (E,E′ and F,F′) and anti-C-terminus (G,G′ and H,H′) FUS IgG antibodies were employed, respectively, to analyze the FUS cellular localization. False coloring and overlays were performed using Adobe Photoshop CS6 software; 15 samples were analyzed with a confocal laser-scanning microscope. Scale bars 10 μm (E–H); 100 μm (E′–H′). MF, morphogenetic furrow.

Flies expressing human FUS in the eye have severe defects such that, externally, compound eyes showed loss of pigmentation and fused ommatidia (Fig. 1Aa). We previously employed those animals to screen the ability of the *Drosophila* FUS (dFUS)-interacting lncRNA *hsrω* to modulate such degeneration and found that its knockdown strongly suppressed the FUS-induced toxicity (Fig. 1Ab) (Lo Piccolo et al., 2017b). The observed rescue combined with a strong reduction of FUS abundance (Fig. 1B–D), the formation of N-terminal FUS fragments (NTF47 and NTF40) (Fig. 1B) and an alteration of FUS solubility, with the level of soluble FUS being reported to be very low upon depletion of *hsrω* (Lo Piccolo et al., 2017b). The decline of FUS, together with its fragmentation, suggested an involvement of a protease in the degradation of FUS. We examined this possibility as below. In physiological conditions, the RBP FUS is mainly enriched in nuclei and its abnormal distribution into cytoplasmic compartment is associated with diseases. In this regard, when expressed in flies, both cytoplasmic and nuclear FUS were detected (Fig. 1E,E′,G,G′). Curiously, the antibody raised against an N-terminal FUS epitope immunoreacted only with nuclear FUS species, while cytoplasmic FUS was revealed only by using an antibody raised against a C-terminal epitope (Fig. 1E,E′,G,G′). Knocking down the lncRNA *hsrω* caused a dramatic change in the FUS localization with the FUS being exclusively observed in nuclei (Fig. 1F,F′,H,H′). The reason why we observed a different immunoreactivity is unknown. We conclude that knocking down the lncRNA *hsrω* has multiple effects on FUS, and that both the reduction of FUS abundance and the prevention of cytoplasmic localization are critical events underlying the suppression of toxicity.

### The lncRNA *hsrω* regulates the methylation of FUS by controlling DART5

Since the arginine methylation of FUS is known to control its cellular localization and/or its solubility (Dormann et al., 2012; Hofweber et al., 2018; Qamar et al., 2018), we next aimed to assess whether the lncRNA *hsrω* could involve the regulation of such a PTM.

To investigate whether the RBP FUS is methylated when expressed in flies and whether the RNAi (IR is used to indicate knockdown) of *hsrω* affects such regulation, we conducted immunoprecipitation experiments with the monoclonal anti-FUS antibodies employing total proteins extracted from adult heads before and after knockdown of *hsrω*. The amount of immunoprecipitated FUS (IP^FUS^) with monomethylated arginine (MMA) and dimethylated arginine (DMA) was then evaluated by western blotting with a pan-MMA and a pan-DMA antibody, respectively. Given the dramatic reduction of FUS abundance caused by *hsrω* RNAi, to obtain a significant quantity of IP^FUS^ upon depletion of *hsrω*, we needed to employ a higher amount of total protein. As such, we were further able to qualitatively compare the level of the pan-MMA and pan-DMA signals among the diverse samples.

**Fig. 2.**
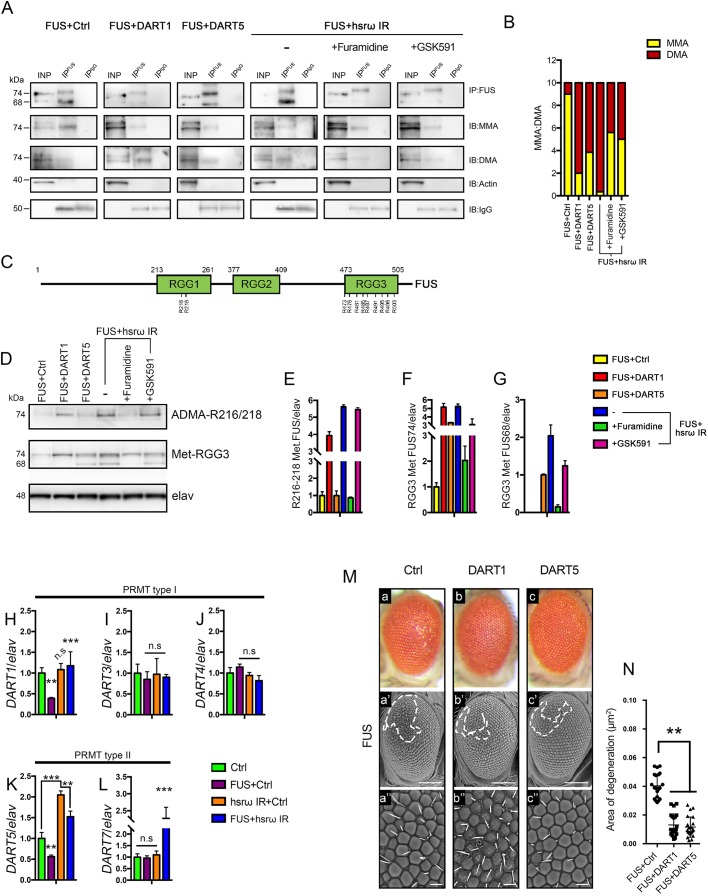
**A decline of arginine methyltransferase activity is critical** **for** **FUS toxicity in *Drosophila*.** (A) The anti-C-terminus FUS IgG was used to immunoprecipitate FUS (IP^FUS^) from total proteins extracted from the adult heads of newly eclosed flies of genotype *GMR-GAL4/+;*UAS*-FUS/+;*UAS*-GFP IR/+* (FUS+Ctrl) and *GMR-GAL4/+;*UAS*-FUS/+;*UAS*-hsrω IR/+* (FUS+hsrω IR), respectively, raised at 25°C. Western blots with anti-C-terminus FUS IgG antibody were performed as a control (IP:FUS). Anti-pan monomethylated (MMA) and -pan dimethylated (DMA) arginine IgG antibodies were used to screen for the final level of arginine methylation of FUS (IB:MMA and IB:DMA, respectively). Anti-Actin (IB:Actin) and anti-IgG (IB:IgG) were used as controls. The methylation status of FUS was also examined upon genetical and pharmacological manipulation of DART1 and DART5 activity. DART1 and DART5 were overexpressed in flies carrying *GMR-GAL4/+;*UAS-*FUS/+;*UAS-*DART1/+* (FUS+DART1) and *GMR-GAL4/+;*UAS-*FUS/*UAS-*DART5;+* (FUS+DART5), respectively. The DART activity was impaired in FUS+hsrω IR flies fed with 25 μM of furamidine (a PRMT1 inhibitor) and GSK591 (a PRMT5 inhibitor), respectively, throughout their development as shown by the reduced intensity of the IB:DMA bands. (B) Gel plots were obtained with ImageJ32 software and used to measure the relative abundance of MMA and DMA in each fraction. (C) The figure depicts the distinct FUS domains and marks the arginine (R) to be methylated in RGG1 and those that might be similarly modified in RGG3. (H–L) Total mRNAs were extracted from the eye discs of L3 larvae at three independent times and further analyzed by qRT-PCR in triplicate. The expression of both genes encoding type I PRMTs (*DART1*, *DART3* and *DART4*) (C–E) and II PRMTs (*DART5* and *DART7*) (F–G) were normalized to the level of *elav*. The transcript abundance is the mean of nine independent reactions for each fly line. **P<0.01, ***P<0.001; n.s., not significant. The genotypes were as follows: *GMR-GAL4/+;+;*UAS*-GFP IR/+* (Ctrl), *GMR-GAL4/+;*UAS-*FUS/+;*UAS*-GFP IR/+* (FUS+Ctrl), *GMR-GAL4/+;+;*UAS-*hsrω IR/*UAS-*GFP IR* (hsrω IR+Ctrl) and *GMR-GAL4/+;*UAS-*FUS/+;*UAS-*hsrω IR/+* (FUS+hsrω IR). (M) Light and scanning electron micrographs of the external eye surface of flies of genotypes, a–a″, *GMR-GAL4/+;*UAS-*FUS/+;*UAS-*lacZ/+* (FUS+Ctrl); b–b″, *GMR-GAL4/+;*UAS-*FUS/+;*UAS-*DART1/+* (FUS+DART1) and c–c″, *GMR-GAL4/+;*UAS-*FUS/*UAS-*DART5;+* (FUS+DART5) raised at 25°C. Lower panels show a higher magnification. Anterior is to the left and dorsal to the top. The white dashed line highlights the area of degeneration. Scale bars: 100 μm (middle panels); 50 μm (lower panels). (N) The external eye structure of 100 newly eclosed male flies from each above fly lines were examined under a dissection microscope, and the most representative were analyzed using SEM. The area of degeneration of ∼18 individuals were measured by ImageJ software and reported as µm^2^. **P<0.01.

We found that FUS is methylated when expressed in flies, with MMA-FUS having a higher abundance than DMA-FUS (Fig. 2A,B, FUS+Ctrl). In contrast, we observed a marked shift in the methylation of FUS upon knockdown of *hsrω* with the DMA-FUS the most abundant species identified (Fig. 2A,B, FUS+hsrω IR). Since we performed a canonical immunoprecipitation by omitting the crosslinking reaction and we did not neutralize the eluted immunoprecipitates, the slight electrophoretic shift of the FUS 74 kDa (FUS74) band observed in all the IP^FUS^ lanes may depend on the presence of the co-eluted heavy and light IgG chains and on the different pH between input (INP) and IP.

The mammalian PRMT type I and II enzymes generate intermediate MMA species that are further modified to asymmetrical- (ADMA) and symmetrical-dimethyl arginine (SDMA), respectively (Bedford and Clarke, 2009). Specifically, PRMT1, 3, 4, 6 and 8, as PRMT type I enzymes, catalyze ADMA formation, whereas the type II PRMT5 catalyzes SDMA formation (Bedford and Clarke, 2009; Blanc and Richard, 2017; Gary and Clarke, 1998). Arginine methyltransferases have also been identified in *Drosophila* and have biochemical properties similar to those of mammalian homologs (Boulanger et al., 2004; Jäckel et al., 2015; Scaramuzzino et al., 2015; Wang and Li, 2012).

To confirm that *hsrω* was indeed a player in the FUS arginine methylation, we further examined the level of the signals from two specific anti-methylated FUS antibodies before and after *hsrω* knockdown (Fig. 2D–F, FUS+Ctrl and FUS+hsrω IR). The anti-ADMA R216/218 antibody is raised against the FUS asymmetric DMA residues at the positions 216 and 218, while the antibody Met-RGG3 targets both asymmetric and symmetric FUS dimethylarginine residues (A/SDMA) within the 31 amino acids of the RGG3 domain (Fig. 2C). In line with the above results, we observed the levels of both ADMA R216/218 and Met-RGG3 were significantly higher upon depletion of *hsrω* (Fig. 2D–F, FUS+Ctrl and FUS+hsrω IR). These findings clearly show that *hsrω* can regulate such PTMs of FUS and also indicate that the RNAi of *hsrω* causes FUS to be variously methylated at multiple sites. Notably, the 68 kDa form of FUS (FUS68) could only be detected by the anti-MetRGG3 antibody, suggesting that there are at least two distinct FUS methylated species in the flies.

Given that the accumulation of MMA-FUS has been proposed to be a result of impaired PRMT activity (Suarez-Calvet et al., 2016), we next assayed the transcript abundance of both type I and type II *Drosophila* arginine methyltransferases (ARTs, herein denoted DARTs) in the heads of adult flies (Fig. 2H–L). Flies express nine DARTs of which DART1, DART3, DART4, DART5 and DART7, are the putative homologs of PRMT1, PRMT3, PRMT4, PRMT5 and PRMT7, respectively, while DART8 is the functional homolog of PRMT6 (Anne et al., 2007; Boulanger et al., 2004). Interestingly, *DART1*, *DART5* and *DART7* were all markedly upregulated in adult heads of flies co-expressing the *hsrω* dsRNA and FUS (FUS+hsrω IR). The expression of *DART3* and *DART4* were not affected (Fig. 2I,J). We were not able to detect any *DART2*, *DART6*, *DART8* or *DART9* transcripts, which is in line with the modENCODE mRNA sequencing in this tissue, which shows none of them are expressed in the heads of adult flies (http://www.flyatlas.org/). Of note, knockdown of the lncRNA *hsrω* on its own caused a significant increase only in the *DART5* transcript (Fig. 2K, hsrω IR+Ctrl) while no effect on the levels of other DARTs was identified.

To evaluate whether the upregulation of DART5 alone was indeed able to directly modulate the methylation of FUS, we conducted co-immunoprecipitation (co-IP) experiments using flies co-expressing FUS and DART5. We found that the co-expression of FUS with DART5 significantly affected the methylation of FUS, which would be likely to reduce the intermediate MMA-FUS species and markedly raise up the DMA-FUS levels, the same effect seen with *hsrω* knockdown (Fig. 2A,B, FUS+DART5). As such, we also found that the Met-RGG3 FUS was strongly increased upon the expression of DART5 and again, showed a similar pattern to what was seen upon *hsrω* knockdown (Fig. 2D,F,G, FUS+DART5). As predicted for type II PRMTs, with the ability to symmetrically dimethylate arginine residues, DART5 did not cause any effects on the level of ADMA FUS R216/218 (Fig. 2D,E, FUS+DART5). This result strongly suggest that DART5 symmetrically dimethylates at least one of the nine arginine residues of the FUS RGG3 domain *in vivo* (Fig. 2C).

To understand the functional importance of a DART5-dependent dimethylation of FUS, we further analyzed the external morphology of the compound eyes for flies co-expressing FUS and DART5, and found a significant amelioration of FUS toxicity upon expression of DART5 (Fig. 2Mc,c′,N, FUS+DART5).

Total RNA was extracted from the heads of adult flies and examined in order to confirm the expression of DART5 was modulated as expected (Fig. S1C). Importantly, we found that the transcription of *FUS* was not affected by the overexpression of DART5 (Fig. S1E), thus, confirming that the suppression of FUS toxicity observed above (Fig. 2Mc,c′) was not due to an alteration of the transgenic expression system.

**Fig. 3.**
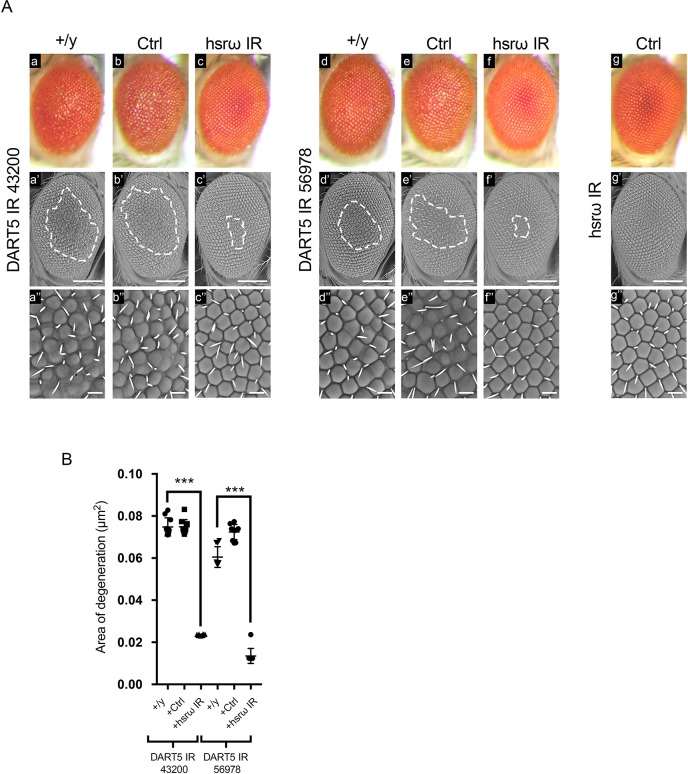
**lncRNA *hsrω* and DART5 functionally** **interact.** (A) Light and scanning electron micrographs of adult compound eyes of flies of genotype (a–a″) *GMR-GAL4/+;*UAS-*DART5 IR 43200/+;+* (DART5 IR 43200++/y), (b–b″) *GMR-GAL4/+;*UAS-*DART5 IR 43200/+;*UAS-*GFP IR/+* (DART5 IR 43200+Ctrl), (c–c″) *GMR-GAL4/+;*UAS-*DART5 IR 43200/+;*UAS-*hsrω IR/+* (DART5 IR 43200+*hsrω IR*), (d–d″) *GMR-GAL4/+;*UAS-*DART5 IR 56978/+;+* (DART5 IR 56978 +/y), (e–e″) *GMR-GAL4/+;*UAS-*DART5 IR 56978/+;*UAS-*GFP IR/+* (DART5 IR 56978 Ctrl), (f–f″) *GMR-GAL4/+;*UAS-*DART5 IR 56978/+;*UAS-*hsrω IR/+* (DART5 IR 56978+ hsrω IR), and (g–g″) *GMR-GAL4/+;+;*UAS-*hsrω IR/*UAS-*GFP IR* (hsrω IR Ctrl) raised at 28°C. Anterior is to the left and dorsal to the top. The white dashed lines highlight the area of degeneration. Scale bars: 100 μm (middle panels); 50 µm (lower panels shown at a higher magnification). (B) The external eye structure of 100 newly eclosed male flies from each of the above fly lines were examined under a dissection microscope, and the most representative were analyzed using scanning electron microscopy. The area of degeneration of ∼10 individuals were measured by ImageJ software and reported as µm^2^. ***P<0.001. Statistical analysis was performed using GraphPad Prism 7.0 software.

**Fig. 4.**
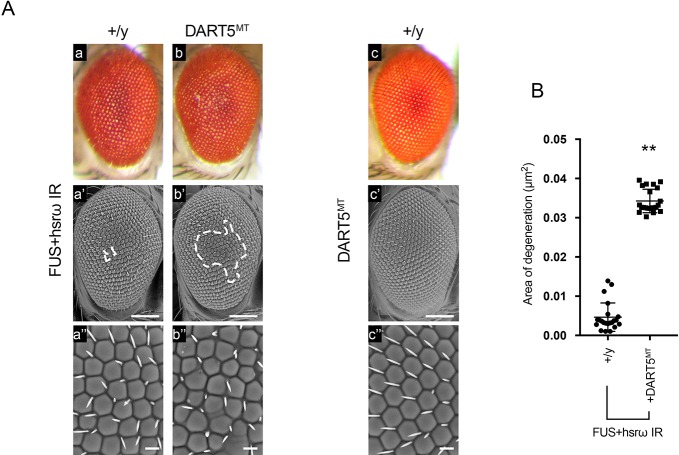
**A loss of DART5 function abolishes the suppressive effect of *hsrω* RNAi on FUS toxicity.** (A) Light and scanning electron micrographs of the external eye surface of flies of genotype (a–a″) *GMR-GAL4/y;*UAS*-FUS/+;*UAS*-hsrω IR*/+ (FUS+*hsrω* IR +/y), (b–b″) *GMR-GAL4/+;*UAS*-FUS/DART5^MT^;*UAS*-hsrω IR/+* (FUS+*hsrω* IR DART5^MT^), and (c–c″) *GMR-GAL4/+;DART5^MT^;+* (DART5^MT^ +/y) raised at 28°C. Anterior is to the left and dorsal to the top. The white dashed lines highlight the area of degeneration. Scale bars: 100 μm (middle panels); 50 μm (lower panels shown at a higher magnification). (B) The external eye structure of 100 newly eclosed male flies from each above fly lines were examined under a dissection microscope, and the most representative were analyzed using scanning electron microscopy. The area of degeneration of 20 individuals were measured by ImageJ software and reported as µm^2^. Statistical analysis was performed using GraphPad Prism 7.0 software. **P<0.01.

These results suggested that the upregulation of DART5 upon *hsrω* knockdown could play a critical role in the suppression of FUS toxicity. Given a genetic interaction of DART5 and *hsrω* was not reported before, to verify our hypothesis, we next performed an *in vivo* RNAi screen in which we reduced the expression of *DART5* in the fly eyes. Knocking down DART5 caused significant damage to the external eye structure, which could be prevented by the co-depletion of *hsrω* (Fig. 3). Therefore, we showed that *hsrω* functionally interacts with DART5. However, it was unclear whether such interaction was required to drive the suppression of FUS toxicity observed upon knockdown of *hsrω*. To address this question we employed a loss of DART5 function mutant, which has been previously generated and characterized (Gonsalvez et al., 2006). Interestingly, we found that DART5 haploinsufficiency was able to strongly abolish the *hsrω* RNAi-dependent suppression of FUS toxicity (Fig. 4). Taken together, these results reinforce the hypothesis that the upregulation of DART5 was a critical step upon the knockdown of *hsrω* to suppress the toxicity of FUS.

### An impairment of arginine methyltransferase activity is a key factor of FUS toxicity in *Drosophila*

To clarify whether the accumulation of MMA-FUS was due to an impairment in the activity of DARTs, we also examined *DART* transcript abundance upon expression of FUS. Compared to control, FUS-expressing flies showed indeed significant lower levels of *DART1* and *DART5*, respectively (Fig. 2H,K, FUS+Ctrl). No significant difference was observed in the transcripts of *DART3*, *DART4* and *DART7* (Fig. 2I,J,L). These results suggest that FUS exerts a negative effect on the expression of *DART*s, which may have a role in the accumulation of MMA-FUS. We have shown above that overexpression of DART5 is able to ameliorate the FUS toxicity, therefore we also examined whether other DARTs likewise have similar effects (Fig. 2M,N). We found that overexpressing DART1 significantly improved the eye defects caused by FUS (Fig. 2Mb,b′,N, FUS+DART1) to a similar level to what is seen upon expression of DART5. We confirmed the expression of DARTs to be modulated as expected by performing quantitative (q)RT-PCR (Fig. S1). Of note, we found that the expression of DART1, as above shown for DART5, also did not affect the transcription of *FUS*. Moreover, knocking down both the type I and II DARTs caused no effects on the FUS toxicity (Fig. S2) and the modulation of the expression of DARTs on their own did not affect the eye integrity (Fig. S3). Co-immunoprecipitation (co-IP) experiments were also conducted and revealed that the expression of DART1 caused a significant shift in the methylation of FUS, with the DMA-FUS levels markedly increased, similar to what was seen with DART5 (Fig. 2A,B, FUS+DART1). In addition, the levels of ADMA R216/218 were strongly enhanced as expected (Fig. 2D,E, FUS+DART1). Interestingly, we also observed a markedly increase in the Met-RGG3 immunoreactivity to suggest that DART1 was able to asymmetrically modify FUS at multiple sites (Fig. 2D,F,G, FUS+DART1).

Taken together, these results strongly support the hypothesis that an impairment of arginine methyltransferase activity caused by excessive levels of FUS is a key factor underlying the accumulation of toxic MMA-FUS in *Drosophila*.

### Arginine dimethylation of FUS by DART1 and DART5 is the fundamental modification underlying the *hsrω* knockdown-dependent suppression of FUS toxicity

**Fig. 5.**
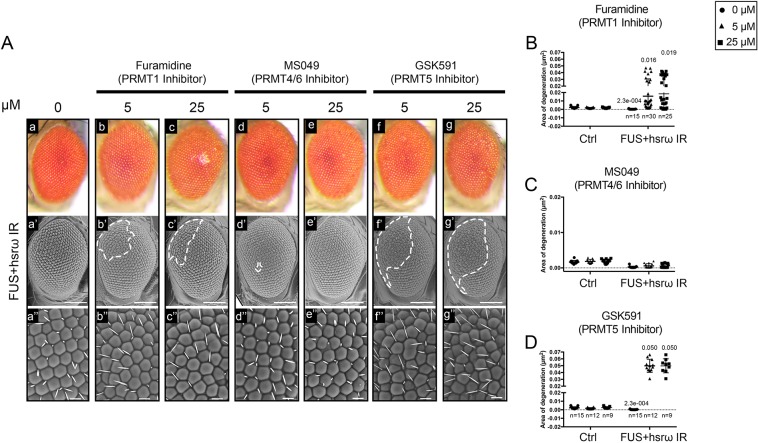
**DART1- and DART5-mediated arginine dimethylation of FUS are fundamental modifications underlying the *hsrω* RNAi-dependent suppression of FUS toxicity.** (A) The effect of arginine methyltransferase activity was examined by the study of the external eye surface of flies of genotype *GMR-GAL4/+;*UAS*-FUS/+;*UAS-*hsrω IR/+* (FUS+hsrω IR) fed with with 0 (a), 5 and 25 μM of Furamidine dihydrochloride (b,c), MS049 oxalate salt (d,e), and GSK591 dihydrochloride (f,g), respectively, throughout their development. Light and scanning electron micrographs are shown. Middle panels (scale bar 100 μm). Lower panels show a higher magnification (scale bar: 50 μm). Anterior is to the left and dorsal to the top. The white dashed lines highlight the area of degeneration. (B–D) The eye phenotypes of 100 newly eclosed male flies from each line were examined under a dissection microscope, and the most representative were analyzed using SEM. The area of degeneration of ∼20 eyes of FUS+hsrω IR flies raised at each above condition were measured by ImageJ software and reported as µm^2^. *n*, number of individuals examined. For each drug assayed, the area of degeneration of 18 eyes of control flies carrying *GMR-GAL4/+;+;*UAS-*GFP IR* (Ctrl) were shown (light and scanning electron microscopy micrographs of Ctrl flies are shown in Fig. S4).

To confirm the role of DART activity on the *hsrω* knockdown-dependent suppression of FUS toxicity, we employed chemical inhibitors of arginine methyltransferase to feed larvae co-expressing FUS and *hsrω* dsRNA and, further, we examined the effects of such inhibition on the external eye surface of adult flies. The furamidine, the MS049 and the GSK591 are shown to exhibit selective inhibitory activity against PRMT1, PRMT4/6 and PRMT5 in mammalian cells, respectively. We tested different doses of such drugs and found that both 5 and 25 µM did not affect, on their own, the eye compounds of adult control flies (Fig. S4) instead, either furamidine or GSK591 showed a remarkable abolishment of *hsrω* knockdown-dependent suppression of FUS toxicity (Fig. 5A–D). We did not observe dosage-dependent effects of furamidine or GSK591 since both drugs caused a similar area of degeneration at both concentrations (Fig. 5B,D). Compared to control flies, no effects were observed by the use of MS049 (Fig. 5C). In order to confirm the pharmacological inhibition of arginine methylation of FUS, we performed co-IP experiments using flies fed with furamidine and GSK591 (Fig. 2A,B). Compared to the methylated-FUS species identified in flies fed without drugs, we observed a strong reduction of DMA-FUS and a significant increase of MMA-FUS by the use of furamidine and GSK591, respectively (Fig. 2A,B, FUS+hsrω IR with or without drugs). Moreover, the immunoreactivity of both ADMA-R216/218 and Met-RGG3 were significantly reduced, accordingly (Fig. 2D–G, FUS+hsrω IR with or without drugs). Therefore, both genetic (Fig. 4) and pharmacological (Fig. 2A and Fig. 5A–D) inhibition of arginine methyltransferases have been to impair the *hsrω* modifier activity on FUS toxicity. These results confirmed that the arginine dimethylation of FUS is a key step driven by the knockdown of *hsrω* that suppresses the FUS-dependent neurodegeneration.

### DART1 and DART5 play different roles on FUS physiology

We further aimed to understand the biological significance of the DART1- and DART5-mediated arginine dimethylation of FUS in the mechanisms underlying both the FUS toxicity and its *hsrω* knockdown*-*dependent suppression. Since we and others observed that an abnormal cellular distribution of FUS causes abnormalities in neurons and, here, we show that both DART1 and DART5 have the ability to rescue the FUS toxicity, we next examined whether these arginine methyltransferases play a role in the FUS cellular localization.

**Fig. 6.**
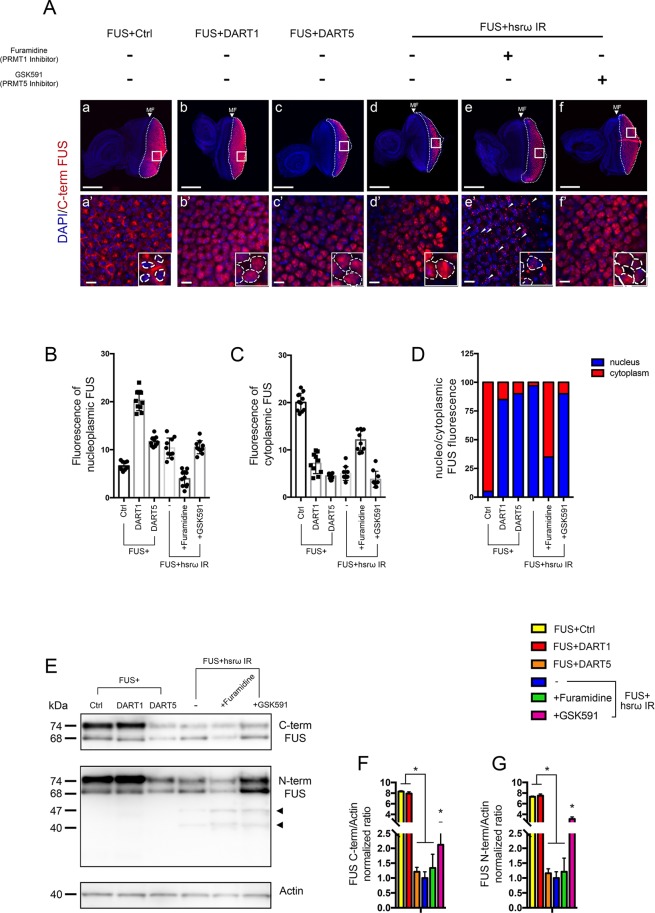
**DART1- and DART5-mediated arginine** **dymethylation play different roles in FUS physiology.** (A) Immunohistochemistry with the anti-C-terminal FUS IgG antibody was performed to study the effects of DART1- and DART5-dependent arginine dimethylation on FUS localization by a comparison of FUS cellular distribution in eye discs of L3 larvae of genotype (b,b′) *GMR-GAL4/+;*UAS-*FUS/+;*UAS-*DART1/+* (FUS+DART1) and (c,c′) *GMR-GAL4/+;*UAS-*FUS/*UAS-*DART5;+* (FUS+DART5) in comparison to control flies (a,a′) of *GMR-GAL4/+;*UAS-*FUS/+;*UAS-*GFP IR/+* (FUS+Ctrl). To gain insight into the role of methylation on FUS localization, FUS immunohistochemistry was also performed in dissected eye discs of L3 larvae of genotype *GMR-GAL4/+;*UAS-*FUS/+;*UAS-*hsrω IR/+* (FUS+hsrω IR) raised at 28°C with 0 (d,d′) and 25 μM of Furamidine dihydrochloride (e,e′) and GSK591 (f,f′), respectively. 15 eye imaginal discs were examined for each genotype. Boxes highlight the area of magnification shown in the lower panels. Insets in lower panels show a 2 times digital magnification of each panel. Dashed lines mark the area of nuclei. The DAPI staining of DNA is shown in blue. FUS staining is shown in red. False coloring and overlays were performed using Adobe Photoshop CS6 software. Scale bars: 50 μm (upper panels), 10 μm (lower panels). (B–D) Quantification of the fluorescence intensity of nucleoplasmic (B) and cytoplasmic FUS (arbitrary units) (C), and the ratio of nucleoplasmic to cytoplamsic FUS (D). (E–G) Total protein was extracted from adult heads and FUS expression was assayed by anti-C-terminal and anti-N-terminal FUS IgG antibodies, respectively. Actin was loaded as internal control to quantify the relative abundance of the major FUS 74 kDa band. Black arrowheads highlight the N-terminal FUS fragments. Statistical analysis was performed by three independent western blots experiments. *P<0.05. The images showing the FUS immunohistochemistry with FUS+GFP IR (a,a′) and FUS+hsrω IR (d,d′) tissues are the same as Fig. 1E–H and are here used as controls to compare the FUS localization upon the genetical and pharmacological modulation of DART activities.

Interestingly, we showed that enhancing the expression of DART1 or DART5 caused FUS to shift to the nuclei (Fig. 6Aa–c). In turn, the inhibition of DART1 activity by the administration of furamidine markedly abolished the recovery of FUS nuclear localization prompted by *hsrω* knockdown and caused FUS to be mainly distributed in the cytoplasm as round-shaped particles (Fig. 6Ad–e,B–D). Quantification of the proportion of nucleoplasmic and cytoplasmic FUS among the different lines was also undertaken (Fig. 6B–D). Of note, the modulation of DART1 did not show any effects on FUS abundance (Fig. 6E–G, FUS+DART1). These results were consistent with the previously reported function of arginine methylation to modulate the FUS cellular localization (Jäckel et al., 2015; Suarez-Calvet et al., 2016).

In contrast, the inhibition of DART5 activity by the GSK591 treatment did not show effect on the nuclear retrieval of FUS upon *hsrω* knockdown (Fig. 6Ad,f,B–D). However, we observed that modulation of DART5 markedly affected the FUS abundance. Indeed, overexpressing DART5 caused a strong decline in the amount of FUS, while its pharmacological inhibition induced a FUS augmentation (Fig. 6E–G, FUS+DART5). Since we showed that the modulation of DART5 expression did not affect the level of *FUS* transcript (Fig. S3F), the underlying mechanism is more likely to be post transcriptional. Therefore, these findings suggest that DART1- and DART5-mediated arginine dimethylation of FUS play different roles in *Drosophila*, with DART1 required to modulate the FUS localization and DART5 being somehow involved in the control of FUS amount.

In consideration of the ability of DART1 to ameliorate the FUS toxicity by promoting FUS nuclear localization without having any effect on the high FUS abundance, these findings also provide a mechanistic insight into FUS toxicity in *Drosophila* and raise the possibility that neurons may tolerate exacerbated levels of nuclear FUS, while high levels of abnormally cytoplasmic distributed FUS are more likely to be detrimental.

Furthermore, we did not observe the cleavage of FUS N-terminal fragments (NTFs) upon expression of DART5 (Fig. 6E) unlike what was found upon *hsrω* knockdown (Fig. 1B and Fig. 6E, arrowheads). Based on these findings, we assumed that lncRNA *hsrω* has the ability to affect the physiology of FUS through several pathways, of which the pathway involving DART5 is not required for FUS cleavage. Moreover, given the rescue of FUS with or without the formation of NTFs, the cleavage of FUS to give NTFs was more likely to have no role on the suppression of FUS toxicity.

Altogether, these results highlight a mechanism for how the lncRNA *hsrω* modifies the FUS toxicity such that a low concentration of recovered nuclear FUS is the result of a dual effect of DART1 and DART5. Indeed, the knockdown of *hsrω* transcriptionally activated DART5 and post-transcriptionally enhanced the function of a proportion of the PRMT type I like DART1 to cause the dimethylation of FUS at multiple sites. The output of such modification was the prevention of FUS localization to the cytoplasm, together with its degradation. In addition, a decline of FUS was also beneficial for the suppression of its negative effect on DART1 to eventually contribute to a nuclear localization.

### DART5-dependent dimethylated FUS is degraded by the proteasome

We pursued the study of DART5-dependent arginine dimethylation in the regulation of FUS turnover.

New evidence has revealed that the human homolog of DART5 (PRMT5) is able to symmetrically dimethylate (sDMA) FUS on the R218 residue (Chitiprolu et al., 2018). Tudor domains bind dimethylated arginine (Bedford and Clarke, 2009; Côté and Richard, 2005) and the survival of motor neuron (SMN) is a Tudor domain-containing protein. Interestingly, SMN was reported to interact with and link the sDMA-FUS to the autophagy receptor p62 (also known as SQSTM1) to cause a selective degradation of FUS aggregates by a so-called C9ORF72–SMN–p62 complex (Chitiprolu et al., 2018). Homologs of SMN and p62 have been identified in *Drosophila* but, despite similar structural and biochemical functions (Avila et al., 2002; Imlach et al., 2012; Nezis et al., 2008), their role in a selective autophagy of RBP aggregates have been never examined. A missense mutation in the *smn* gene was previously characterized to cause a loss of SMN function in flies (Chan et al., 2003; Rajendra et al., 2007). Therefore, given the occurrence of an sDMA-dependent degradation of FUS by the SMN–p62 complex in mammals, we employed such a SMN mutant to test the possibility of a similar mechanism occurring in our models.

We found that the heterozygous SMN^73A0^ mutation was not able to functionally interact with the *hsrω* RNAi-dependent suppression of FUS toxicity. Indeed, we did not observe alteration of the rescued eye morphology nor significant variations of FUS abundance in the flies examined (Fig. S5Aa,b,C). The SMN^73A0^ did not cause toxicity on its own, nor did it affect FUS-expression-mediated eye degeneration (Fig. S5Ac–e,C).

Similarly, we examined a possible involvement of *Drosophila* p62 [Ref(2)P]. We first verified the insertion of a P-element to cause a disruption of *Ref(2)P* transcript (Fig. S6B) and next we assayed the effect of the heterozygous Ref(2)P^KG00926^ mutant on flies co-expressing FUS and *hsrω* dsRNA (Fig. S6A–D). Here, we also did not observe modification of the suppression of FUS toxicity upon the heterozygous loss of Ref(2)P function (Fig. S6Aa,b). The Ref(2)P mutant did not show toxicity on its own (Fig. S6Ae) and unexpectedly, slightly ameliorated the FUS eye degeneration (Fig. S6Ac,d).

The loss of SMN and/or Ref(2)P functions was expected to be likely to affect the rescued eye morphology and/or increase the FUS levels. Given both SMN^73A0^ and Ref(2)P^KG00926^ mutants were not able to modulate the decline of FUS abundance seen upon *hsrω* RNAi expression, these results suggest that the mechanism underlying the degradation of toxic FUS in mammalians may be different in *Drosophila* and the selective autophagy of FUS by the SMN–p62 complex may not have a role in the *hsrω*-knockdown mediated rescue of FUS-mediated toxicity. However, these results did not exclude that the haploinsufficiency by the heterozygous mutations were inadequate to cause an arrest of the hypothetical selective autophagy of FUS. Nevertheless, our findings are consistent with previous reports showing that genetic and pharmacological inhibition of autophagy do not impair the *hsrω*-knockdown mediated rescue of FUS toxicity (Lo Piccolo et al., 2017b). Of note, flies do not express any functional homolog of C9ORF72, which in mammalians has a key role in the complex to cause selective autophagy of FUS aggregates as above mentioned.

**Fig. 7.**
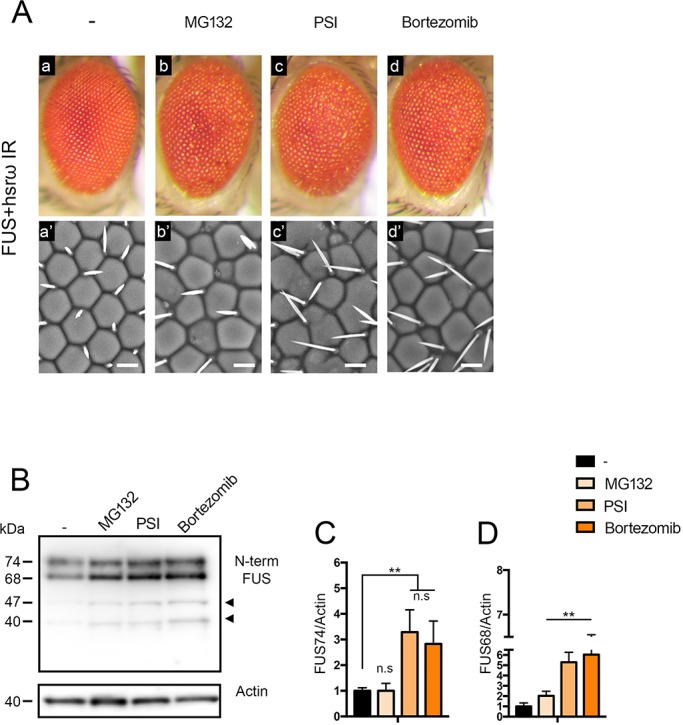
**A *hsrω*-dependent network leads to the degradation of dimethylated FUS via proteasome.** (A) The external eye surface of flies of genotype *GMR-GAL4/+;*UAS-*FUS/+;*UAS-*hsrω IR/+* (FUS+hsrω IR) raised at 28°C were analyzed by light and scanning electron microscopy (a) and compared with those of flies fed with proteasome inhibitors: (b) 50 μM of MG132, (c) 50 μM Proteasome inhibitor I (PSI) and (d) 5 μM Bortezomib, respectively. Scale bars: 50 μm. (B–D) Total proteins were extracted from adult heads and the level of FUS was determined though western blotting with the anti-N-terminal FUS IgG antibody. Actin was used as internal control to quantify the relative abundance of the two major bands (FUS74 and FUS68). Statistical analysis was performed on three independent western blots experiments. **P<0.01, n.s., not significant. Black arrowheads highlight the N-terminal FUS fragments.

Next, we considered the possibility that the proteasome is involved in the degradation of DART5-dependent dimethylated FUS. Therefore, we fed larvae with several drugs including MG132, Proteasome Inhibitor I (PSI) and Bortezomib at different concentrations throughout their development. We observed each drug to have a dosage-dependent effect such that eye morphology of the offspring showed signs of degeneration upon an increase of their concentration (Fig. 7A; Fig. S7A). To monitor the activity of MG132, PSI and Bortezomib in our experimental conditions, we studied the level of ubiquitin by western blotting. The occurrence of poly-ubiquitylated proteins was dosage dependent and confirmed the impairment of 26S proteasome to be as expected (Fig. S7B). We next assessed whether FUS levels were altered following proteasome inhibition. Indeed, compared to control, proteasome-impaired flies showed a significant increase of FUS abundance, with PSI and Bortezomib showing the strongest effect on the inhibition of FUS degradation (Fig. 7B,C). These results indicate that the proteasome is required to degrade FUS upon knockdown of *hsrω*. Moreover, we found that the abundance of NTF47 and NTF40 were also increased upon the use of each drug tested, with a similar trend to what was seen for FUS74 (Fig. 7B, arrowheads). Therefore, these results also suggest that the formation of FUS NTFs is not dependent on the proteasomal activity and confirmed that their generation instead may involve different pathways than that mediated by *hsrω* and DART5 to control the FUS turnover.

## DISCUSSION

### Novel regulatory role of the lncRNA *hsrω*

In this study, we identified a new function of *hsrω* that extends the repertoire of its multiple activities and explains how a nuclear lncRNA can modulate RBPs through regulation of arginine methyltransferases.

Together with the functions of arginine methylation to shape the chromatin and safeguard the chromosome stability in *Drosophila* and mammalians (Ancelin et al., 2006; Bandyopadhyay et al., 2012; Dacwag et al., 2007; Gonsalvez et al., 2006; Kirino et al., 2009; Lacroix et al., 2008; Nishida et al., 2009; Sanchez et al., 2010; Tee et al., 2010), the activity of PRMTs has been also recognized as an important regulator of several aspects of RBPs, including their tendency to undergo to liquid–liquid phase transition (Hofweber et al., 2018; Qamar et al., 2018) and form RNP granules (Guo et al., 2018; Molliex et al., 2015; Purice and Taylor, 2018; Suarez-Calvet et al., 2016; Yoshizawa et al., 2018).

Despite the importance of the type I and II PRMTs, there is still a poor understanding in both *Drosophila* and mammalians of how their gene expression is regulated and how they eventually affect the activity of RBPs.

In this study, we have taken advantage of *Drosophila* models of RBP-associated toxicity to identify that a nuclear lncRNA is a transcriptional regulator of DART5 and post-transcriptional regulator of some type I PRMTs, such as DART1. We further characterized the activity of such methyltransferases to modulate both the cellular localization and homeostasis of the ALS/FTD-causative RBP FUS.

Interestingly, we showed in our fly system that the dimethylated FUS species created through the action of DART5 was eliminated via the proteasome, while in mammalians dimethylation of FUS by PRMT5 has been shown to be necessary for the clearance of FUS aggregates by the C9ORF72–SMN–p62 complex (Chitiprolu et al., 2018). As such, the function of the type II PRMT-dependent arginine methylation of FUS may be conserved among species to determine a decline of excessive level of FUS. However, the underlying mechanism of such an outcome seems to be different given that it requires distinct complexes.

The proteasome is known to efficiently attack toxic aggregation-prone proteins as long as they remain in a soluble state thus, given its involvement in the degradation of dimethylated FUS in *Drosophila*, we assumed that the DART5-mediated PTM may be likely to modulate the phase transition of FUS to facilitate the action of proteasome. In line with this hypothesis, hyper-methylated FUS has been found to be soluble and dispersed while, the hypo-methylated forms were found as aggregates in fibril-like structures (Qamar et al., 2018).

This is the first time that a lncRNA has been shown to ‘supervise’ the activity of a RBP by the regulation of its post-translational modifications.

### Mechanisms of toxicity induced by excessive levels of FUS

Mutations in both coding and non-coding regions of FUS have been reported to cause ALS. Indeed, the majority of mutations are clustered in the C-terminal region that contains a nuclear localization signal (NLS) and lead to an abnormal cytosolic FUS accumulation (Dormann et al., 2010; Gal et al., 2011; Vance et al., 2013). In addition, mutations in the 3′-UTR have been found to increase levels of FUS (Dini Modigliani et al., 2014; Sabatelli et al., 2013). Of note, FUS levels are known to be autoregulated by a mechanism conserved in both mammalians and *Drosophila* in which the human FUS downregulates the endogenous FUS at mRNA and protein levels (Jäckel et al., 2015; Kamelgarn et al., 2018; Lo Piccolo et al., 2017b; Zhou et al., 2013). Recently, it has been proven that loss of such autoregulatory mechanism leads to a gain of FUS function that alters both RNA metabolism and the autophagy–lysosome pathway (Ling et al., 2019). These findings confirmed what we and other groups have previously reported, showing that the FUS toxicity strongly correlates with its expression level.

In addition, in this study we found that the expression of FUS also has negative effects on the transcription of several DARTs, including DART5 which, importantly, we showed had a critical role in modulating FUS abundance. Therefore, our data suggest that high levels of FUS may trigger a vicious circle, such that the negative effect of FUS on type II arginine methyltransferase causes a reduction of FUS degradation and may contribute to altering its auto-regulatory mechanism.

Furthermore, we showed that FUS also downregulates the transcript level of DART1, which is a protein that in turn can also modify FUS. In this study, we produced evidence to show that for correct nuclear FUS localization, FUS needs to be dimethylated by the activity of DART1. Therefore, the decline of DART1 activity causes an increase of FUS in the cytosolic compartments. These findings add to our hypothesis that the alteration of the autoregulatory mechanism is itself responsible for the loss of FUS nuclear localization, particularly given its negative effect on DART1.

Nonetheless, whether the DART1-dependent FUS dimethylation reduces the export of FUS to the cytoplasm, or by contrast, facilitates its nuclear import still remains an open question. Of note, FUS is imported into the nucleus by the activity of the protein transportin (TNPO1) (Dormann et al., 2010). Methylation of arginine residues within the RGG domain of FUS by PRMT1 has been reported to weaken the FUS–TNPO1 binding and, thus, to cause an increase of cytosolic FUS in HeLa cells (Dormann et al., 2010). Therefore, the activity of PRMT1 on FUS was shown to impair the nuclear translocation of FUS and enhance the FUS toxicity (Dormann et al., 2012; Tradewell et al., 2012). In contrast, we observed that the expression of DART1 caused a retrieval of FUS so that it had a nuclear distribution. Based on this evidence, we assumed that such a modification may have a different outcome in *Drosophila* that in fact facilitates the nuclear import of FUS. It will be interesting to investigate whether the possible different effects of methylation of FUS by DART1 and the human homolog PRMT1 may depend on a different arginine residue being targeted or on the combination of diverse methylated residues.

Taken together, these data extend the view on the mechanisms of FUS toxicity. Through the regulatory activity on arginine methyltransferases, FUS seems to be ‘in charge of its own destiny’, because indeed, it can influence two of the most critical aspects of its physiology that, interestingly, have roles in the diseases in which it is involved, namely, its abundance and its cellular localization.

## MATERIALS AND METHODS

### Fly stocks

Flies were raised and maintained on standard cornmeal-agar-yeast-based food at 25°C. The fly line *GMR-GAL4* was described previously (Takahashi et al., 1999). The transgenic fly lines bearing the UAS*-*human *FUS* (FUS) or the UAS*-hsrω* dsDNA (Bloomington, 59616) transgenes have been described previously (Ishiguro et al., 2017; Mallik and Lakhotia, 2009). Flies bearing +;UAS*-GFP IR* (9330), +;UAS-*lacZ* (1776), +;UAS*-DART1 IR* (31348), +;UAS*-DART4 IR* (36833), UAS*-DART5 IR/*Cyo*;+* (43200), +;UAS*-DART7 IR* (36832), UAS*-DART5 IR;+* (56978), +;SMN^73A0^/TM6B,Tb (4802), Ref(2)P^KG^/Cyo;+ (13287) were obtained from the Bloomington Stock Center. The flies bearing +;UAS*-myc-DART5* and DART5-1;+ (loss-of-function mutant, hereafter referred to as DART5^MT^) were kindly provided by Prof. Gregory M. Matera (Department of Biology, University of North Carolina) and were described in Gonsalvez et al., (2006). Flies bearing +;UAS-*DART1* (F003189) were obtained from FlyORF collection. To minimize the effects of genetic background, the flies used in this study were backcrossed six times to the *w*^1118^ strain.

### Light microscopy and scanning electron microscopy

Newly eclosed flies were anesthetized with 99% diethyl ether, and the external surfaces of the eyes of corresponding flies were analyzed with the Olympus SZX10 stereomicroscope. Images were taken with a coupled Olympus DP22 microscope digital camera. In the examination using a scanning electron microscope, after anesthesia, adult flies were mounted on stages and inspected under the scanning microscope TM-1000 (Hitachi) in the low vacuum mode. Area showing fused ommatidia was defined as degenerated. Degenerated area was marked and measured by the ImageJ32 software. When not specified, 15 adult flies of each phenotype were analyzed. Statistical analysis was performed with GraphPad Prism 7.0.

### Protein extraction and western blotting

*Drosophila* adult flies were frozen in liquid nitrogen, and the heads were separated by vigorous vortexing for 45 s. Crude extracts were obtained by homogenization of 10 heads in 100 µl SDS Sample buffer 2X containing 125 mM Tris-HCl pH 6.8, 20% glycerol, 4% SDS, 0.01% bromophenol blue dye (BPB), 10% 2-β-mercaptoethanol (2-ME) with Biomasher II (Funakoshi). Homogenized heads were centrifuged at 15,000×***g*** at 4°C for 20 min. A 5 µl of cleared crude extracts were run on a 5–20% gradient polyacrylamide gel (ATTO, E-T/R/D520 L) and then transferred onto a Transblot-Turbo membrane (Bio-Rad). The membrane was blocked with PVDF blocking reagent (Toyobo) at room temperature and then incubated overnight with mouse monoclonal anti-FUS antibody (against the C-terminus, Santa Cruz Biotechnology, 4H11; 1:1000), rabbit polyclonal anti-FUS antibody (N-terminus, Bethyl A300-302; 1:5000), mouse monoclonal anti-asymmetric dimethylarginine FUS R216, R218 (Funakoshi, clone 2B12; 1:1500), rat monoclonal anti-methylated RGG3 FUS (Merck, 9G6; 1:1000), rat monoclonal anti-elav (DSHB, 7E8A10; 1:750), mouse monoclonal anti-actin antibody (clone AC-40, Sigma-Aldrich; 1:5000) or mouse monoclonal pan-ubiquitin (Ubi-1) (Abcam, ab7254; 1:1000). After overnight incubation, the membranes were incubated with HRP-conjugated secondary antibodies (1:20,000). Primary and secondary antibodies were diluted with ‘Can get signal’ solution I and II, respectively (Toyobo). Membranes were then treated with Super-Signal West Dura chemiluminescent substrate (Thermo Fisher Scientific), and images were taken and analyzed with an Amersham Imager 600 (GE Healthcare Life Sciences).

### Co-immunoprecipitation

Adult heads were dissected as above and protein extraction was carried out by use of IP lysis buffer pH 7.4 (25 mM Tris-HCl, 150 mM NaCl, 1 mM EDTA, 1% NP40 and 5% glycerol) containing cOmplete™, EDTA-free Protease Inhibitor Cocktail (Sigma). Immunoprecipitation of FUS was performed with the monoclonal anti-FUS antibody (C-terminus, Santa Cruz, 4H11; 1:1000) by Pierce^TM^ Crosslink Magnetic PI/Co-IP Kit (Thermo Fisher Scientific). The IP beads were then collected, according to the manufacturer's instruction and eluted in elution buffer pH 2.0 for a subsequent western blotting assay. To study the FUS arginine methylation, we employed the mouse monoclonal anti-pan monomethyl arginine IgG antibody (Abcam, 16B11; 1:400) and the mouse monoclonal anti-pan dimethyl arginine IgG antibody (Abcam, 21C7; 1:400). The monoclonal anti-actin antibody (clone AC-40, Sigma-Aldrich) (1:5000) and the mouse IgG isotope (Invitrogen, 10400C) (1:5000) were used as control.

### Immunohistochemistry and confocal imaging

Immunohistochemistry of wander third-instar larvae (L3) was performed as described in Lo Piccolo et al. (2017b). Briefly, L3 eye disc dissection carried out in 1× Phosphate-buffered saline (PBS). Tissues were further fixed in ice-cold 4% paraformaldehyde at 25°C for 30 min, washed in PBS containing 0.3% Triton X-100 (PBT) four times for 10 min each, and blocked with 2% normal goat serum (NGS) at 25°C for 30 min. Samples were next incubated with the following primary antibodies: mouse monoclonal anti-FUS antibody (against the C-terminus, Santa Cruz Biotechnology, 4H11; 1:50), rabbit polyclonal anti-FUS antibody (against the N-terminus, Bethyl A300-302) (1:1000) at 4°C for 16 h with agitation, and, after washing with PBT 5 times 10 min each, they were treated with the Alexa Fluor 488-conjugated anti-rabbit IgG secondary antibodies (1:400, Invitrogen) and Alexa Fluor 594-conjugated anti-mouse IgG (1:400, Invitrogen) at 25°C for 2 h. All primary and secondary antibodies were diluted in PBT-2% NGS. The Hoechst 33258 Pentahydrate (bis-Benzimide) (Thermo Fisher Scientific) was used to stain the nuclei. Samples were mounted in Vectashield (Vector Laboratories Inc.) and observed under a confocal laser-scanning microscope (OLYMPUS Fluoview FV10i). Images were analyzed with MetaMorph Imaging System 7.7 (Molecular Devices Inc.). Using ImageJ (v1.48, NIH), an outline was drawn around each cell and circularity, area, mean fluorescence measured, along with several adjacent background readings. The total corrected fluorescence (TCF)=integrated density–(area of selected cell×mean fluorescence of background readings), was calculated. False coloring and overlays were performed using Adobe Photoshop CS6 software.

### RNA extraction and qRT-PCR

RNA was isolated from either eye imaginal discs or adult heads as follows. Three replicates of 50 eye imaginal discs from third-instar larvae or three replicates of ten adult heads were collected for each genotype. Homogenization was performed with a 1-ml syringe with a 27-G needle. Total RNA was isolated using an RNeasy Lipid Tissue Mini-Kit (Qiagen), followed by a DNase treatment (DNase I, Roche), and 0.5 µg of total RNA was reverse transcribed to cDNA using a Prime Script RT reagent kit (TaKaRa) according to the manufacturer's instructions. qRT-PCR was performed in triplicate for each single extraction with SYBR Green Master Mix [SYBR Premix Ex Taq II (Tli RNase H Plus; TaKaRa)] using the CFX96 Touch™ Real-Time PCR detection system (Bio-Rad), with the specific primer pairs as listed in Table S1. Data were analyzed with a standard curve-based method, as calculated with CFX Manager™ software. The specificity of primers was tested with melt curves created by CFX Manager™ software and the agarose gel electrophoresis of amplified fragments. The abundance of *elav* or *RpL32* transcript were used as an internal control.

### Solid food feeding assay

Selective chemical inhibition of the type I arginine methyltransferases DART1, DART4 and DART6, and of the type II methyltransferase DART5 was performed with furamidine dihydrochloride (Funakoshi 5202/5), MS049 oxalate salt (Funakoshi 5685/10), GSK 591 dihydrochloride (Funakoshi 5777/10), respectively. A 10 mM stock solution of each compound was freshly prepared in H_2_O and used to dissolve 1.2 g of instant *Drosophila* medium (Carolina Biological Supply Company) at 0, 5 and 25 µM in a final volume of 4.5 ml for each vial.

Proteasome impairment was induced by MG132 (Sigma, M7449), proteasome inhibitor I (PSI) (Millipore, 539160) and Bortezomib (Wako, 179324-69-7), respectively. A 10 mM stock solution of MG132 and PSI were freshly prepared in DMSO, respectively and used to dissolve 1.2 g of instant *Drosophila* medium (Carolina Biological Supply Company) at 0, 5, 25, 50 or 100 µM in a final volume of 4.5 ml for each vial. A stock of Bortezomib solution was obtained in ethanol and used to perform the solid food feeding assay at 0, 1, 5 and 12.5 µM as above. Parental flies were crossed in triplicate in either solvent or drug-based vial at 28°C under controlled humidity and light protection and removed 3 days later. Each drug solution was filled every 3 days according to the selected final concentration. New eclosed flies were observed under the light microscope and, when not differently reported, 15 representative males from at least two independent vials were selected for the external eye structure analysis with a scanning electron microscope.

### Statistical analysis

Statistical analysis was performed in GraphPad Prism 7. When control and treatment groups were handled in parallel, for two-grouped analyses a paired *t*-test was applied. The Mann–Whiney *U*-test was used to compare data between two independent groups. The Kruskal–Wallis test, followed by Dunnett's multiple comparison test were used to statistically analyze differences among three or more groups. The significance of differences between the variables was shown based on the *P*-value obtained (not significant when *P*>0.05; *P<0.05; **P<0.005; ***P<0.001). Values are presented as a mean and error bars indicate the standard deviation (s.d.).

## Supplementary Material

Supplementary information
