# Supplementary material for: The lncRNA hsrω regulates arginine dimethylation of human FUS to cause its proteasomal degradation in Drosophila
Source: J Cell Sci. 2019 Oct 23;132(20):jcs236836. doi: 10.1242/jcs.236836 (PMC6826006; doi:10.1242/jcs.236836)
Supplement: Supplementary information [file joces-132-236836-s1.pdf]

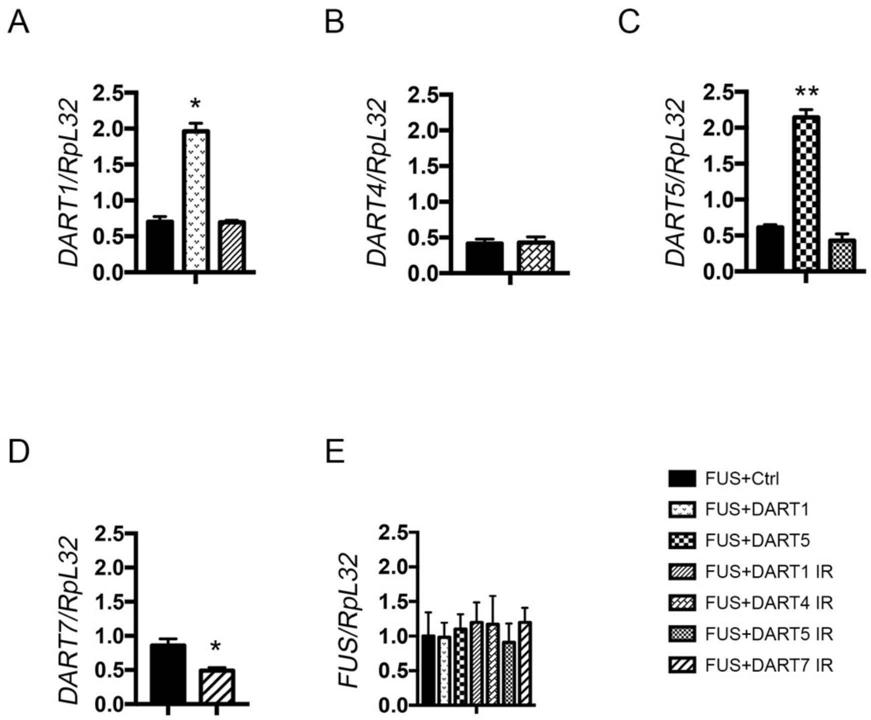


**FIGURE S1 Modulation of DARTs gene expression has no effects on FUS transcription.** RNAs were extracted at three independent times from eye discs of L3 larvae carrying *GMR-GAL4/+;*UAS-*FUS/+;*UAS-*lacZ/+* (FUS+Ctrl), *GMR-GAL4/+;*UAS-*FUS/+;*UAS-*DART1/+* (FUS+DART1), *GMR-GAL4/+;*UAS- *FUS/*UAS-*DART5; +* (FUS+DART5), *GMR-GAL4/+;*UAS-*FUS/+;*UAS-*DART1 IR/+* (FUS+DART1 IR), *GMR-GAL4/+;*UAS-*FUS/+;*UAS-*DART4 IR/+* (FUS+DART4 IR), *GMR-GAL4/+;*UAS-*DART5 IR 43200/+;+* (FUS+DART5 IR), *GMR-GAL4/+;*UAS-*FUS/+;*UAS-*DART7 IR/+* (FUS+DART7 IR). The expression of DART1 (**A**), DART4 (**B**), DART5 (**C**), DART7 (**D**) were studied by qRT-PCR to confirm their modulation to be as expected. The gene expression of each DARTs is normalized respect that in FUS+Ctrl, respectively. (**E**) The abundance of *FUS* was similarly assayed. The transcripts were normalized to the level of *RpL32*. The transcript abundance is the average of six independent reactions for each fly line. Statistical analysis were performed using GraphPad Prism 7.0 software. n=6, * p <0.05, ** p <0.01.

Journal of Cell Science • Supplementary information

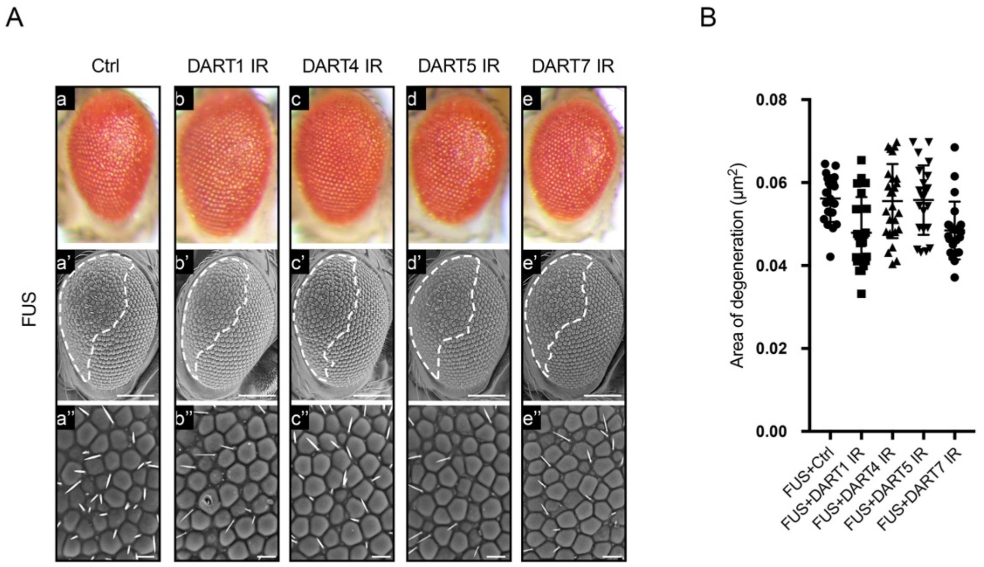


**FIGURE S2 The RNAi of arginine methyltransferases type I and II has no effect on FUS toxicity**

**A)** Light and scanning electron micrographs (SEM) of the adult compound eyes of flies carrying **a-a'')** *GMR-GAL4/+;*UAS-*FUS/+;*UAS-*lacZ/+* (FUS+Ctrl), **b-b'')** *GMR-GAL4/+;*UAS-*FUS/+;*UAS-*DART1 IR/+* (FUS+DART1 IR), **c-c'')** *GMR-GAL4/+;*UAS-*FUS/+;*UAS-*DART4 IR/+* (FUS+DART4 IR), **d-d'')** *GMR-GAL4/+;*UAS-*FUS/*UAS-*DART5 IR 43200/+;+* (FUS+DART5 IR), **e-e'')** *GMR-GAL4/+;*UAS-*FUS/+;*UAS-*DART7 IR/+* (FUS+DART7 IR) raised at 25°C. Middle panels (scale bar 100 µm). Lower panels show a higher magnification (scale bar: 50 µm). Anterior is to the left and dorsal to the top. The white dot lines highlight the area of degeneration. **B)** The external eye structure of 100 newly eclosed male flies from each above fly lines were examined under a dissection microscope, and the most representative were analysed using SEM. The area of degeneration of about 15 individuals were measured by ImageJ software and reported as µm$^2$. Statistical analysis were performed using GraphPad Prism 7.0 software. n=15

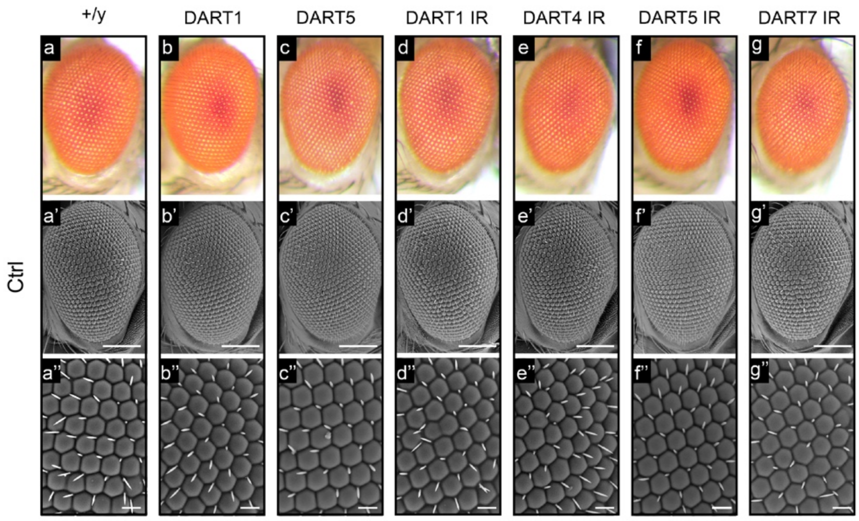


**FIGURE S3 Modulation of arginine methyltransferases type I and II has not detrimental effects on the eye morphology**

**A)** Light and scanning electron micrographs (SEM) of the adult compound eyes of flies carrying **a-a'')** *GMR-GAL4/+;*+;UAS-*lacZ*/+ (Ctrl), **b-b'')** *GMR-GAL4/+;+;*UAS-*lacZ*/UAS-*DART1* (DART1+Ctrl), **c-c'')** *GMR-GAL4/+;* UAS-*DART5/+;*UAS-*lacZ*/+ (DART5+Ctrl), **d-d'')** *GMR-GAL4/+;+;*UAS-*lacZ*/UAS-*DART1 IR* (DART1 IR+Ctrl), **e-e'')** *GMR-GAL4/+;+;*UAS-*lacZ*/UAS-*DART4 IR* (DART4 IR+Ctrl), **f-f'')** *GMR-GAL4/+;*UAS-*DART5 IR 43200/+;*UAS-*lacZ*/+ (DART5 IR+Ctrl), **g-g'')** *GMR-GAL4/+;+;*UAS-*lacZ*/UAS-*DART7 IR* (DART7 IR+Ctrl) raised at 25°C. Middle panels (scale bar 100 µm). Lower panels show a higher magnification (scale bar: 50 µm). Anterior is to the left and dorsal to the top. The white dot lines highlight the area of degeneration.

Journal of Cell Science • Supplementary information

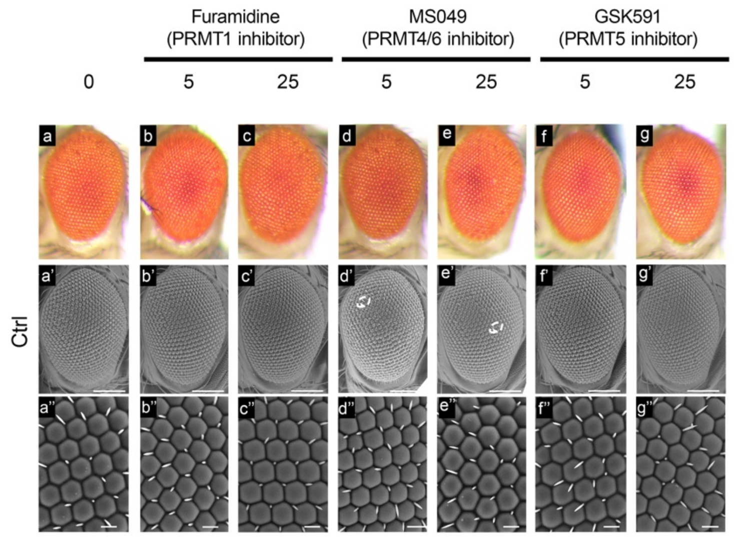


**FIGURE S4 Pharmacological inhibition of DART activity has not toxic effect on control flies.**

The effect of inhibition of arginine methyltransferase activity were examined by the study of external eye surface of control lies carrying *GMR-GAL4/+;*+;UAS-*GFP IR/*+ (Ctrl) fed with with 0 (a), 5 and 25 μM of Furamidine dihydrochloride (b and c), MS049 oxalate salt (d and e), GSK591 dihydrochloride (f and g), respectively throughout their development. Light and scanning electron micrographs (SEM) were shown. Middle panels (scale bar 100 μm). Lower panels show a higher magnification (scale bar: 50 μm). A very small area of degeneration is marked by dot lines.

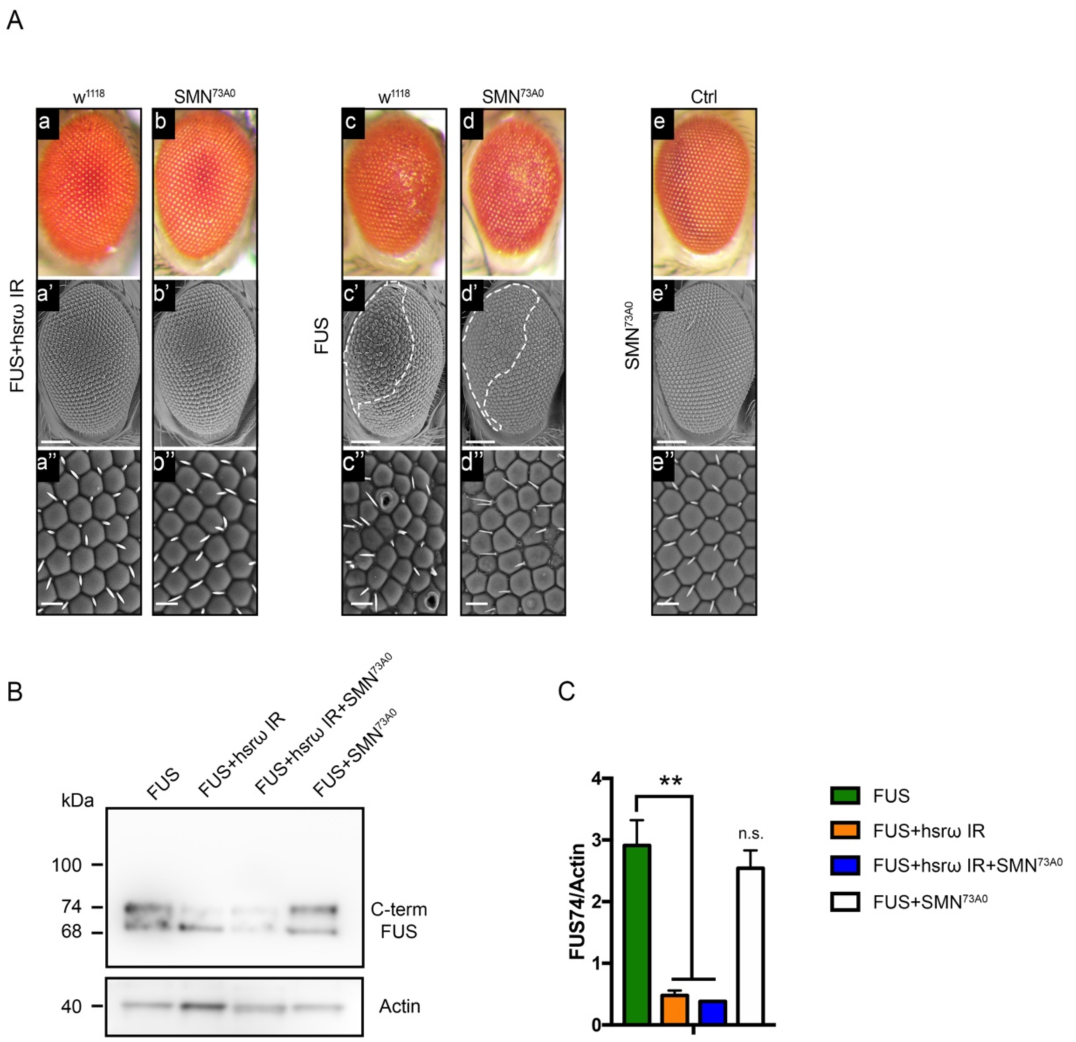


**FIGURE S5 Survival of Motor Neuron (SMN) is not required for the *hsrω*-dependent FUS toxicity rescue.**

**A)** Light and scanning electron micrographs (SEM) of the adult compound eyes of flies carrying **a-a'')** *GMR-GAL4/+;*UAS-*FUS/+;*UAS-*hsrω IR/+* (FUS+hsrω), **b-b'')** *GMR-GAL4/+;*UAS-*FUS/+;*UAS-*hsrω IR/SMN$^{73A0}$* (FUS+hsrω+SMN$^{73A0}$), **c-c'')** *GMR-GAL4/+;*UAS-*FUS/+;+* (FUS) **d-d'')** *GMR-GAL4/+;*UAS-*FUS/+;SMN$^{73A0}$/+* (FUS+SMN$^{73A0}$) **e-e'')** *GMR-GAL4/+;+;SMN$^{73A0}$*/UAS-*lacZ* (SMN$^{73A0}$+Ctrl) raised at 25°C. Middle panels (scale bar 100 µm). Lower panels show a higher magnification (scale bar: 50 µm). Anterior is to the left and dorsal to the top. The white dot lines highlight the area of degeneration. The point mutation G202S in the SMN primary structure causes the loss of SMN function (SMN$^{73A0}$). **B-C)** Total protein were extracted from adult heads of the above flies and FUS expression was assayed by anti-Cterm FUS IgG antibody. Actin was loaded as internal control to quantify the relative abundance of the major FUS 74 kDa band (FUS74). Statistical analysis were performed using GraphPad Prism 7.0 software by three independent western blots experiments. n=3, ** p <0.01, n.s.= not significant.

Journal of Cell Science • Supplementary information

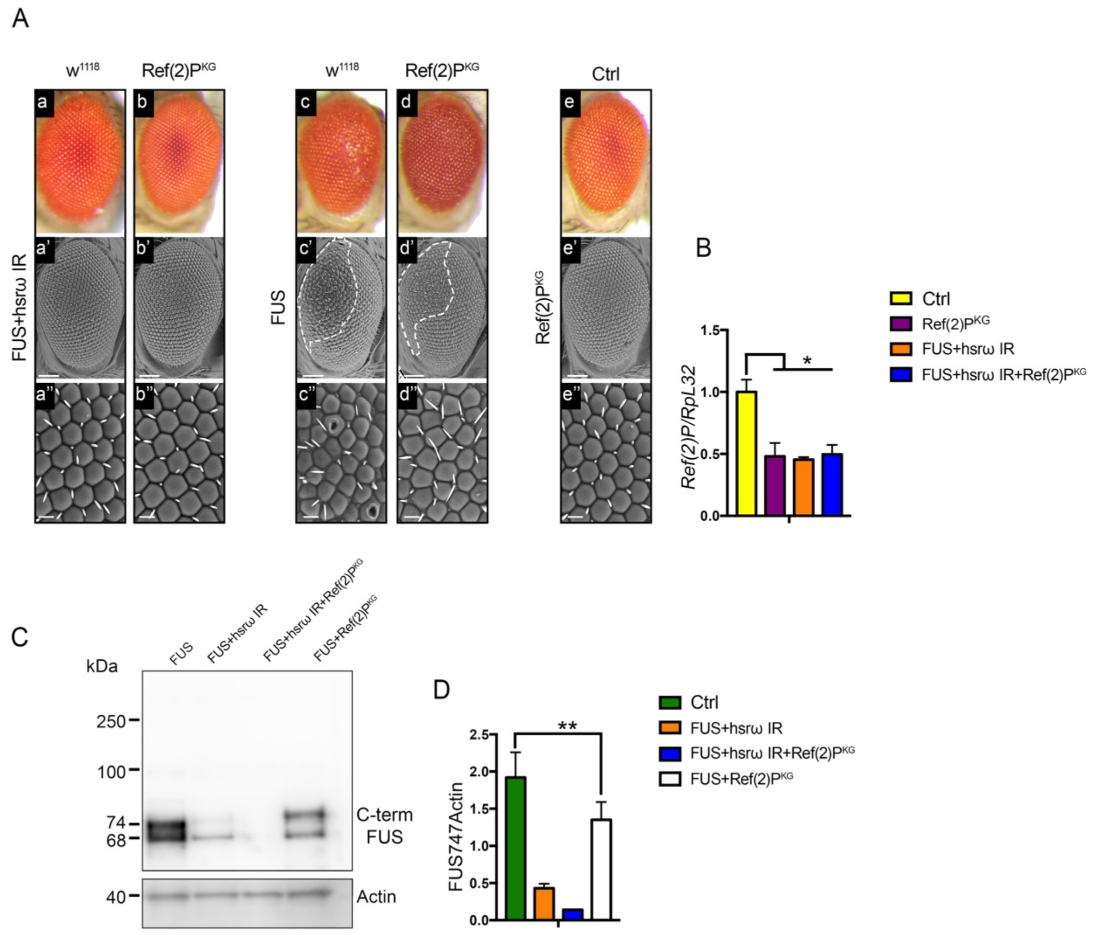


**FIGURE S6 The *Drosophila* p62/Ref(2)P is not required for the *hsrω*-dependent FUS toxicity rescue.**

**A)** Light and scanning electron micrographs (SEM) of the adult compound eyes of flies carrying **a-a'')** *GMR-GAL4/+;*UAS-*FUS/+;*UAS-*hsrω IR/+* (FUS+hsrω), **b-b'')** *GMR-GAL4/+;*UAS-*FUS/Ref(2)P$^{KG}$;*UAS-*hsrω IR/+* (FUS+hsrω+Ref(2)P$^{KG}$), **c-c'')** *GMR-GAL4/+;*UAS-*FUS/+;+* (FUS) **d-d'')** *GMR-GAL4/+;*UAS-*FUS/Ref(2)P$^{KG}$;/+* (FUS+Ref(2)P$^{KG}$), **e-e'')** *GMR-GAL4/+;Ref(2)P$^{KG}$/+;*UAS-*lacZ/+* (Ref(2)P$^{KG}$ +Ctrl) raised at 25°C. Middle panels (scale bar 100 µm). Lower panels show a higher magnification (scale bar: 50 µm). Anterior is to the left and dorsal to the top. The white dot lines highlight the area of degeneration. **B)** RNAs were extracted from the adult heads of the above flies at three independent times and further analysed by RT-qPCR in triplicate to characterized the Ref(2)P$^{KG}$ mutant. The expression of *Ref(2)P* gene was normalized to the abundance of *elav*. A statistical analysis was performed using GraphPad Prism 7.0 software. n=9,* $p < 0.05$, n.s= not significant. **C)** Total protein were extracted from adult heads of the above flies and FUS expression was assayed by anti-Cterm FUS IgG antibody. Actin was loaded as internal control to quantify the relative abundance of the major FUS 74 kDa band (FUS74). Statistical analysis were performed using GraphPad Prism 7.0 software by three independent western blots experiments. n=3, ** $p < 0.01$.

Journal of Cell Science • Supplementary information

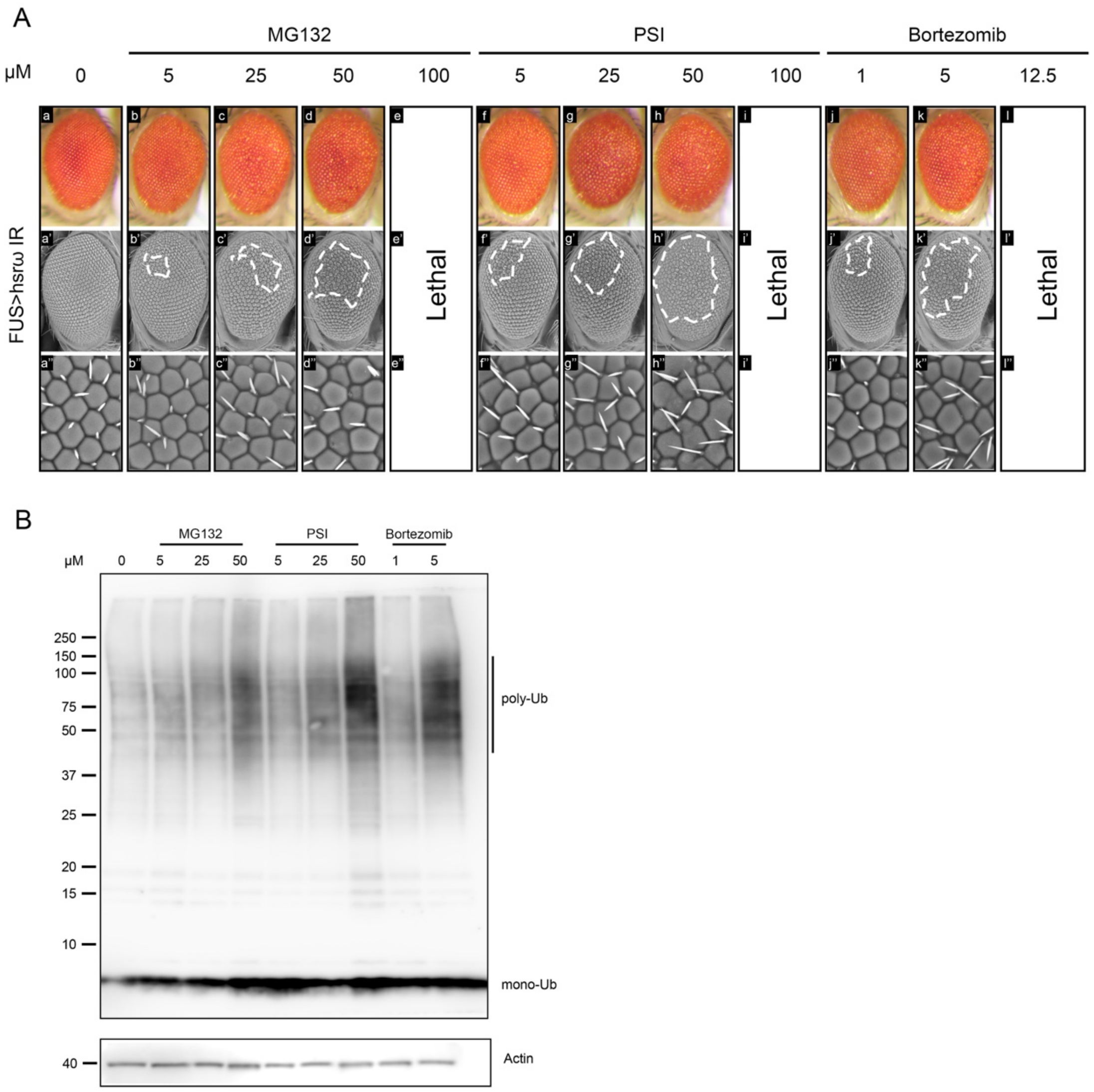


**FIGURE S7 Pharmacological inhibition of proteasome causes the accumulation of poly-ubiquitylated proteins**

**A)** A dosage dependent effect of several proteasome inhibitors were assayed by comparison of the external eye surface of flies carrying *GMR-GAL4*/+*;*UAS-*FUS*/+*;*UAS-*hsrω IR*/+ (FUS+hsrω IR) raised at 28°C with that of flies fed throughout the development with increasing concentration of MG132, Proteasome inhibitor I (PSI) and Bortezomib. **B)** Total proteins were extracted from adult heads of flies raised as above and the ubiquitin expression was assayed by anti-pan ubiquitin IgG antibody. Actin was loaded as internal control. Both the drug employed shown a dosage dependent effect to cause the accumulation of poly-ubiquitylated proteins (poly-Ub).

Journal of Cell Science • Supplementary information

**Table S1 List of primers used in this study**

| Name | Forward Sequence (5'-3') | Reverse Sequence (5'-3') |
|---|---|---|
| *RpL32* | AGATCGTGAAGAAGCGCACC | CGATCCGTAACCGATGTTGG |
| *DART1* | TCACGCCCAATTCAAATGCG | CTTGCCCTGAAACAGATGCTT |
| *DART2* | TCTTCAGGCACAAGACTGTCC | TCACCTGTATAACTCTGCCGAA |
| *DART3* | GATGAGCTAACCACTTGTCTGTT | GGCACTGATCTTTTTGGCTCT |
| *DART4* | GCAGAAGCTAGAGTTTACCAACA | GCGGCTATCATGTACTCCTTG |
| *DART5* | TTTACCATGTCCGACGTGAATG | AGCCGTACCATCAGGTTGC |
| *DART6* | CCGAATGGATGGGAAATGCTC | CTCCACGTTGCACCAAAAGT |
| *DART7* | TCCACAGAGATCCAAGTGGG | CATGGTTGTAGATGCCGATGG |
| *DART8* | GATTGAGGATAACGGCCTGAC | GCAGCAGGTAGAAGCCCATC |
| *DART9* | GAATCTGCTGAGAGAAATGGCG | CCCACGTCCAGGACTATCTTAT |
| *Ref(2)P* | AATCGAGCTGTATCTTTTCCAGG | AACGTGCATATTGCTCTCGCA |
| *elav* | CGCAGCCCAATACGAATGG | CATTGTTTGCGGCAAGTAGTTG |
| *FUS* | CCTGGGCGAGAATGTTACAA | GGCTGTCCCGTCTTCTTATTT |
